# Zinc Piracy as a Mechanism of *Neisseria meningitidis* for Evasion of Nutritional Immunity

**DOI:** 10.1371/journal.ppat.1003733

**Published:** 2013-10-31

**Authors:** Michiel Stork, Jan Grijpstra, Martine P. Bos, Carmen Mañas Torres, Nathalie Devos, Jan T. Poolman, Walter J. Chazin, Jan Tommassen

**Affiliations:** 1 Department of Molecular Microbiology and Institute of Biomembranes, Utrecht University, Utrecht, Netherlands; 2 GlaxoSmithKline Vaccines, Rixensart, Belgium; 3 Department of Biochemistry and Chemistry and Center for Structural Biology, Vanderbilt University School of Medicine, Nashville, Tennessee, United States of America; Faculté de Médecine Paris Descartes, Necker, France

## Abstract

The outer membrane of Gram-negative bacteria functions as a permeability barrier that protects these bacteria against harmful compounds in the environment. Most nutrients pass the outer membrane by passive diffusion via pore-forming proteins known as porins. However, diffusion can only satisfy the growth requirements if the extracellular concentration of the nutrients is high. In the vertebrate host, the sequestration of essential nutrient metals is an important defense mechanism that limits the growth of invading pathogens, a process known as “nutritional immunity.” The acquisition of scarce nutrients from the environment is mediated by receptors in the outer membrane in an energy-requiring process. Most characterized receptors are involved in the acquisition of iron. In this study, we characterized a hitherto unknown receptor from *Neisseria meningitidis*, a causative agent of sepsis and meningitis. Expression of this receptor, designated CbpA, is induced when the bacteria are grown under zinc limitation. We demonstrate that CbpA functions as a receptor for calprotectin, a protein that is massively produced by neutrophils and other cells and that has been shown to limit bacterial growth by chelating Zn^2+^ and Mn^2+^ ions. Expression of CbpA enables *N. meningitidis* to survive and propagate in the presence of calprotectin and to use calprotectin as a zinc source. Besides CbpA, also the TonB protein, which couples energy of the proton gradient across the inner membrane to receptor-mediated transport across the outer membrane, is required for the process. CbpA was found to be expressed in all *N. meningitidis* strains examined, consistent with a vital role for the protein when the bacteria reside in the host. Together, our results demonstrate that *N. meningitidis* is able to subvert an important defense mechanism of the human host and to utilize calprotectin to promote its growth.

## Author Summary

The sequestration of essential nutrient metals is a first line of defense used by vertebrates to limit the growth of invading pathogens, a process termed “nutritional immunity.” As a part of this defense mechanism, neutrophils and other cells produce massive amounts of calprotectin, a protein that limits bacterial growth by chelating Zn^2+^ and Mn^2+^ ions. We demonstrate here that *Neisseria meningitidis*, a resident of the human nasopharynx that occasionally causes sepsis and meningitis, is able to survive and propagate in the presence of calprotectin. *N. meningitidis* responds to zinc limitation by the overproduction of an outer membrane protein, called CbpA, that functions as a receptor for calprotectin and enables the bacteria to utilize calprotectin as zinc source. The ability of *N. meningitidis* to use calprotectin as a zinc source subverts an important defense mechanism of the host and adds a new mechanism to the host-pathogens arms race.

## Introduction

The outer membrane of Gram-negative bacteria functions as a protective barrier against harmful compounds from the environment, including many antibiotics. Most nutrients can pass the outer membrane by passive diffusion via pore-forming proteins, known as porins. However, diffusion can only satisfy the growth requirements if the extracellular concentration of the nutrients is high. The uptake of nutrients that are scarce in the environment or whose sizes exceed the exclusion limit of the porins is mediated by receptors in an energy-requiring process [1]. Energizing these receptors requires an inner-membrane-based proteinaceous machinery known as the TonB complex, which spans the periplasm and couples the energy of the proton gradient across the inner membrane to the transport process in the outer membrane [1], [2].

In the human host, the concentration of free iron is too low to sustain bacterial growth because it is bound by the iron-transport and -storage proteins transferrin and lactoferrin. This defense mechanism of the host is known as nutritional immunity. Pathogenic Gram-negative bacteria have evolved receptor-based mechanisms to cope with iron limitation. Because of their importance for pathogenicity, these iron-acquisition mechanisms have been studied extensively in many bacterial pathogens. How such bacteria transport other essential transition metals, such as zinc and manganese, across the outer membrane is largely unknown. The availability of these metals is also limiting for bacterial growth in the human host, who responds to infection by the production of metal-binding proteins such as calprotectin and metallothioneins [3], [4]. Hence, efficient uptake mechanisms for these metals may constitute important virulence factors.

*Neisseria meningitidis* is a strictly human pathogen. Usually, it resides as a commensal on the mucosal surfaces of the nasopharynx, but occasionally it causes sepsis and meningitis. Based on homology searches, 12 genes encoding TonB-dependent receptors have been identified in the available meningococcal genome sequences [5]. Five of these TonB-dependent family (Tdf) members, LbpA, TbpA, HmbR, HpuB, and FrpB (a.k.a. FetA), have well-defined roles in iron acquisition; they function as (part of the) receptors for lactoferrin, transferrin, hemoglobin, hemoglobin/haptoglobin, and the siderophore enterobactin, an iron-chelating compound produced by *Escherichia coli*, respectively [6]. The expression of these proteins and of the hitherto uncharacterized receptor TdfK is induced under iron limitation [7]. In microarray analyses, the expression of several other *tdf* genes, including *tdfH* and *tdfI*, appeared unresponsive to iron availability [8], [9]. Hence, we considered the possibility that the encoded receptors are involved in the acquisition of essential nutrient metals other than iron. In a previous study, we demonstrated that the expression of *tdfI* is induced under zinc limitation and that the encoded protein is involved in zinc acquisition [10]. This protein is now called ZnuD because of its role in zinc uptake. Here, we characterized another receptor, TdfH (locus tags NMBH4476_0730 and NMB1497 in the genome sequences of strains H44/76 and MC58, respectively), which, because of its function resolved here (*vide infra*), will from now on be called CbpA.

## Results

### Regulation of the expression of CbpA

**Figure 1:**
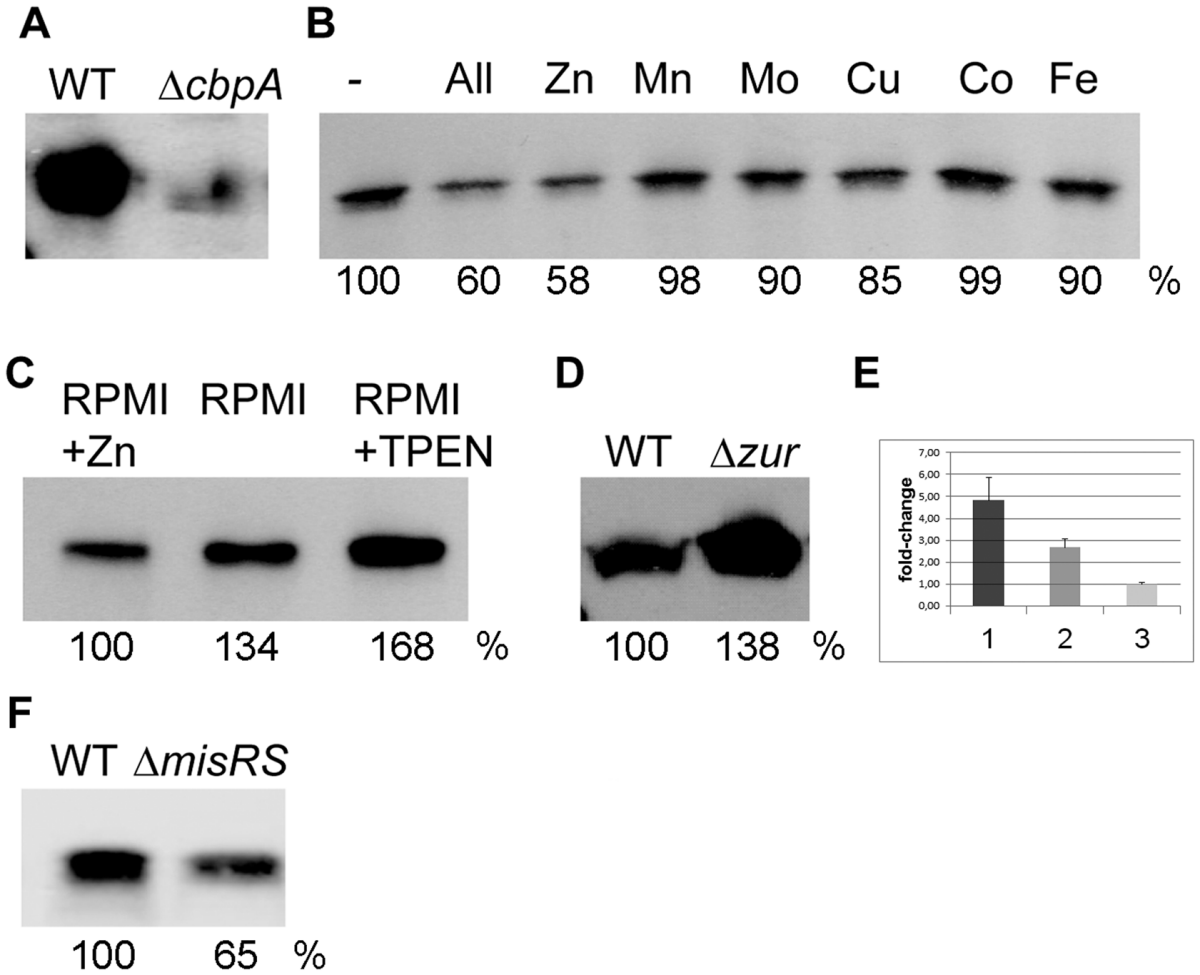
Regulation of *cbpA* expression. (A–D, F) Whole cell lysates were prepared from equal amounts of cells (on OD_550_ basis) and analyzed by SDS-PAGE followed by immunoblotting using antiserum directed against CbpA. In panels B, C, D, and F, the CpbA bands were quantified relative to the first lane on the blots and relative expression levels are indicated underneath the lanes. (A) Wild-type strain HB-1 (WT) and its Δ*cbpA* mutant derivative were grown in RPMI medium. (B) Strain HB-1 was grown in RPMI (-), or on RPMI supplemented with a cocktail of trace metals (All) or with these metals separately. The metal sources used were ZnSO_4_, MnCl_2_, Na_2_MoO_4_, CuSO_4_, CoCl_2_, and FeCl_3_ each at a final concentration of 1 µM. (C) Strain HB-1 was grown in RPMI (middle lane) or in RPMI supplemented with 1 µM ZnSO_4_ or with 0.5 µM of the zinc chelator TPEN. (D) Strain HB-1 (WT) and its Δ*zur* mutant derivative were grown in RPMI supplemented with 0.5 µM ZnSO_4_. (F) Strain HB-1 (WT) and its Δ*misRS* mutant derivative were grown in RPMI supplemented with 0.5 µM ZnSO_4_. (E) Expression analysis of *cbpA* as measured in qRT-PCR experiments. Column 1, *cbpA* expression in strain HB-1 grown in RPMI relative to that in strain HB-1 grown in RPMI supplemented with 0.6 µM ZnSO_4_; Column 2, *cbpA* expression in strain HB-1Δ*zur* relative to that in strain HB-1 both grown in RPMI supplemented with 0.6 µM ZnSO_4_; Column 3, *cbpA* expression in strain HB-1Δ*zur* grown in RPMI relative to that in strain HB-1Δ*zur* grown in RPMI supplemented with 0.6 µM ZnSO_4_.

First, we determined whether we could evaluate CbpA expression on Western blots. To that end, cells of strain HB-1 were grown in RPMI medium, a synthetic medium that is not supplemented with trace elements and therefore has a low concentration of heavy metals [10]. Analysis of the whole cell lysates by SDS-polyacrylamide gel electrophoresis (SDS-PAGE) and immunoblotting revealed that the protein is expressed under those conditions (Figure 1A). We then tested whether the expression of *cbpA* could be repressed by supplementation of the medium with transition metals. The presence in the medium of a cocktail of ZnSO_4_, MnCl_2_, Na_2_MoO_4_, CuSO_4_, CoCl_2_, and FeCl_3_, each at a final concentration of 1 µM, indeed reduced CbpA synthesis (Figure 1B, lane 2). When the metals were tested separately, only zinc reduced CbpA synthesis at a 1-µM concentration (Figure 1B), whilst supplementation of the medium with the zinc chelator N,N,N′,N′-Tetrakis-(2-pyridylmethyl)-ethylenediamine (TPEN) further induced expression of CbpA (Figure 1C). Notably, supplementation of the medium with FeCl_3_ even at a 100-fold higher concentration did not affect CbpA synthesis (Figures 1B and S1), consistent with the lack of responsiveness of *cbpA* expression to iron availability in transcriptome analyses [8], [9].

Expression of zinc-regulated genes is controlled by the regulatory protein Zur, which acts as a repressor under zinc-replete conditions [10], [11]. To determine whether *cbpA* expression is controlled by Zur, strain HB-1 and a *zur*-mutant derivative were grown under zinc-replete conditions and whole-cell lysates were analyzed by SDS-PAGE and Western blotting. Consistent with the observed zinc regulation (Figure 1B), *cbpA* expression was enhanced when the repressor Zur was inactivated (Figure 1D). The zinc-dependent regulation of *cbpA* was further confirmed by quantitative real-time reverse transcription PCR (qRT-PCR), which showed an almost 5-fold repression upon supplementation of the RPMI medium with 0.6 µM ZnSO_4_ (Figure 1E). In agreement with the higher levels of CbpA in the *zur* mutant (Figure 1D), qRT-PCR experiments showed that *cbpA* transcript levels were increased in the *zur* mutant relative to the wild-type strain under zinc-replete conditions (Figure 1E). Furthermore, these transcript levels in the *zur* mutant were unaffected by zinc availability (Figure 1E). Together, these results confirm that *cbpA* expression is regulated by zinc availability and demonstrate that this regulation is mediated by Zur.

Previous microarray analyses suggested that *cbpA* expression is controlled by the MisRS (PhoPQ) system [12], [13], a two-component regulatory system suggested to be involved in the adaptation of *N. meningitidis* to growth on host cells [14]. Consistent with these results, we found that CbpA synthesis is reduced in a *misRS*-deletion mutant of strain HB-1 (Figure 1F). Thus, expression of *cbpA* appears to be under dual control of both Zur and the MisRS two-component system.

### CbpA binds calprotectin

**Figure 2:**
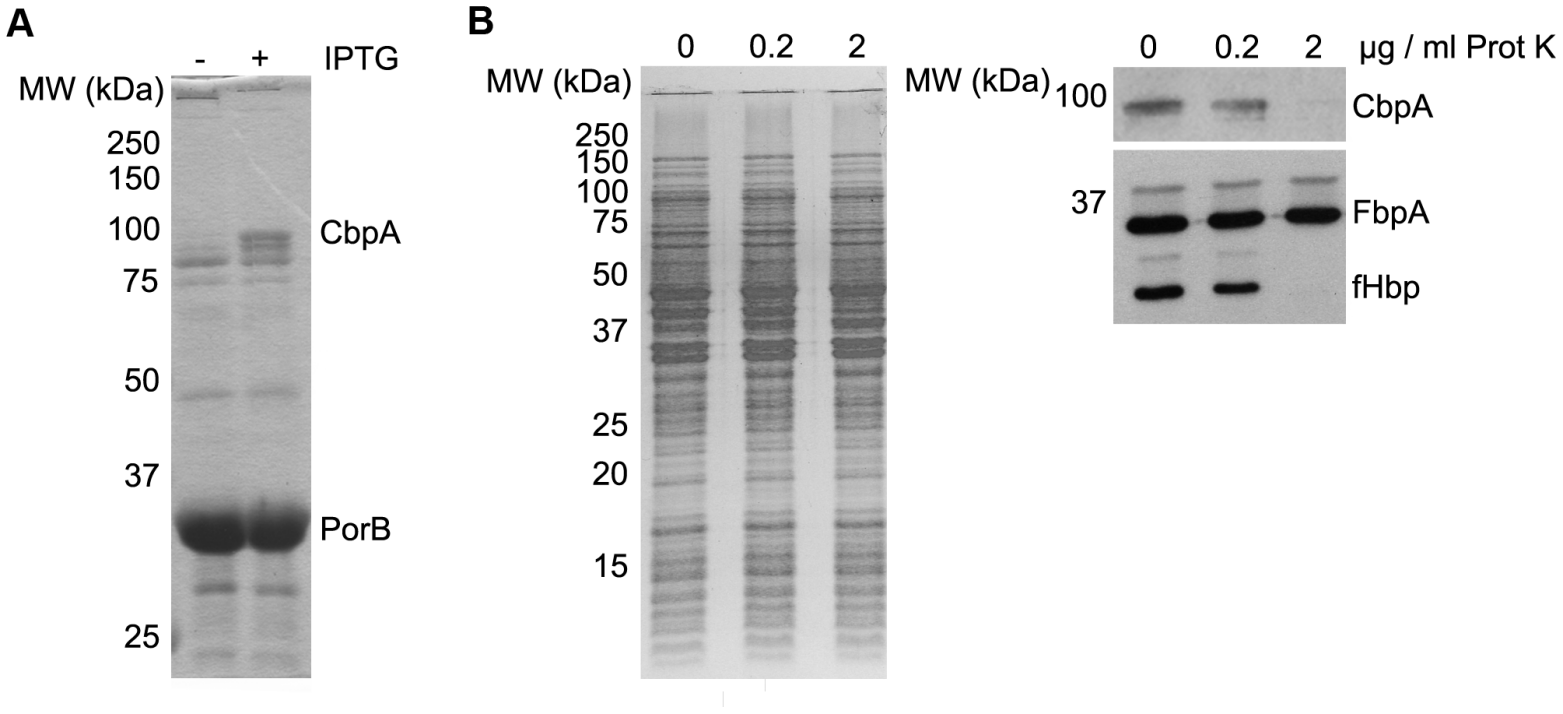
CbpA is a cell-surface-exposed outer membrane protein. (A) OMVs were isolated from cells of strain CE1523 containing pEN-*cbpA* grown in the presence or absence of isopropyl-β-D-thiogalactopyranoside (IPTG) and analyzed by SDS-PAGE. The gel was stained with Coomassie brilliant blue. The position of CbpA is indicated. (B) Intact cells were treated with proteinase K (Prot K) at the concentrations indicated at the top and analyzed by SDS-PAGE followed by staining with Coomassie brilliant blue (left) or immunoblotting (right) with antibodies directed against the proteins indicated. In all panels, the positions of molecular weight marker proteins (MW) are indicated at the left (in kDa).

As a Tdf family member, CbpA is expected to be embedded in the outer membrane as a 22-stranded β-barrel with an N-terminal plug domain that closes the pore in the barrel [1]. The outer membrane localization of CbpA was confirmed by isolating outer membrane vesicles (OMVs) from strain CE1523 containing *cbpA* under *lac*-promoter control on plasmid pEN11-*cbpA* by extracting the cells with deoxycholic acid (DOC). Like the outer membrane marker protein, the porin PorB, CbpA was present in the insoluble OMV fraction (Figure 2A). The cell-surface exposure of CbpA was confirmed in protease-accessibility experiments. Like the cell-surface-exposed lipoprotein fHbp (factor H-binding protein), CbpA was degraded when intact cells were treated with proteinase K, while the periplasmic iron-binding protein FbpA was inaccessible (Figure 2B). Thus, CbpA is a surface-exposed outer membrane protein that is expected to bind a ligand from the environment.

**Figure 3:**
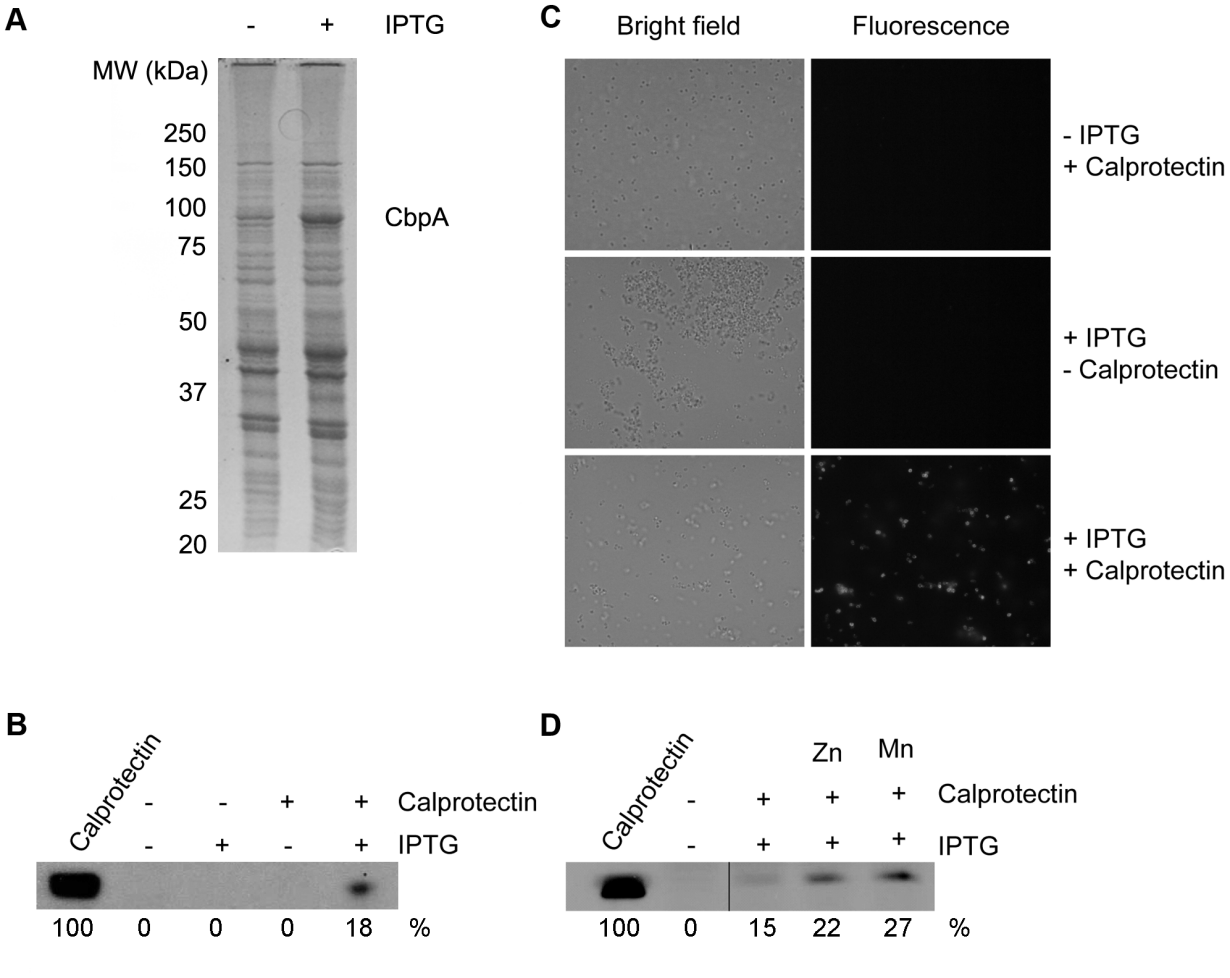
Binding of calprotectin to *N. meningitidis* cells expressing *cbpA*. (A) Cells of strain HB-1Δ*cbpA* containing pEN11-*cbpA* were grown in TSB either with or without IPTG as indicated on the top and the protein patterns of samples of the cells were examined by SDS-PAGE. The position of overproduced CbpA is indicated at the right and those of molecular mass standard proteins (in kDa) at the left. (B) After growth, the cells were harvested, washed and incubated for 1 h with or without calprotectin as indicated. After extensive washing of the cells, whole cell lysates were analyzed by Western blotting with a calprotectin-specific mAb. The first lane contains purified calprotectin for reference. The amounts of calprotectin in the lanes were quantified relative to this reference sample and are indicated at the bottom of the blot. (C) Cells of strain HB-1Δ*cbpA* containing pEN11-*cbpA* were grown, harvested and washed as in panels A and B and then incubated with calprotectin for 15 h. After extensive washing, the cells were successively incubated with the calprotectin-specific mAb and Alexafluor-594-conjugated goat anti-mouse IgG antiserum. After extensive washing, the cells were examined by bright field (left) and fluorescence (right) microscopy. The scale bar represents 10 µM. (D) Similar experiment as in panel B, except that the binding assay in the last two lanes was done in the presence of 1 µg/ml of ZnSO_4_ or MnCl_2_ as indicated.

Based on their molecular masses, Tdf members in various bacteria can be classified in two categories. The smaller ones, with molecular masses of ∼70–75 kDa, are usually involved in the binding of small ligands, such as siderophores. ZnuD and FrpB of *N. meningitidis* belong to this category. The larger ones have molecular masses of ∼100 kDa and bind proteins as ligands. Examples of this category are LbpA and TbpA of *N. meningitidis*, which are involved in the uptake of iron from lactoferrin and transferrin, respectively. The mature form of CbpA of strains H44/76 and MC58, *i.e.* after cleavage of the predicted signal sequence, consists of 896 amino-acid residues and has a predicted molecular mass of 101.2 kDa. Therefore, we predicted that CbpA binds a proteinaceous ligand. One of the putative ligands we considered was calprotectin. Calprotectin is a major protein component in the cytoplasm of neutrophils and is released in abscesses by cell lysis. It limits the growth of invading pathogens by sequestering the essential nutrient metals zinc and manganese [4]. Calprotectin is also produced by stromal cells in the nasopharynx [15], the normal niche of *N. meningitidis*. To determine whether CbpA can bind calprotectin, we incubated cells of a *cbpA* mutant of strain HB-1 containing *cbpA* under *lac*-promoter control on plasmid pEN11-*cbpA* (Figure 3A) with calprotectin. After harvesting and extensive washing of the bacteria, whole cell lysates were analyzed by SDS-PAGE and Western blotting with a monoclonal antibody (mAb) directed against calprotectin. The results showed that the bacteria could bind calprotectin but only if expression of *cbpA* was induced with isopropyl-β-D-thiogalactopyranoside (IPTG) (Figure 3B). Binding of calprotectin to the CbpA-producing cells was confirmed by indirect immunofluorescence microscopy (Figure 3C). These experiments suggested that CbpA is a calprotectin-binding protein and, therefore, the protein is dubbed CbpA.

The calprotectin used in the experiments described above was not deliberately loaded with nutrient metal ions although the protein might have been partially loaded by chelation of metal present as contaminants in the buffer solutions during purification and the binding assays. We next asked whether binding to CbpA-producing cells might be improved if the calprotectin is loaded with key nutrient metal ions. To test this possibility, the binding experiments were repeated in the presence of Zn^2+^ or Mn^2+^ ions. In both cases, the binding of calprotectin to the CbpA-producing cells was enhanced (Figure 3D). Thus, CbpA appears to have a higher affinity for calprotectin that is loaded with its ligands.

### CbpA enables meningococci to use calprotectin as a zinc source

**Figure 4:**
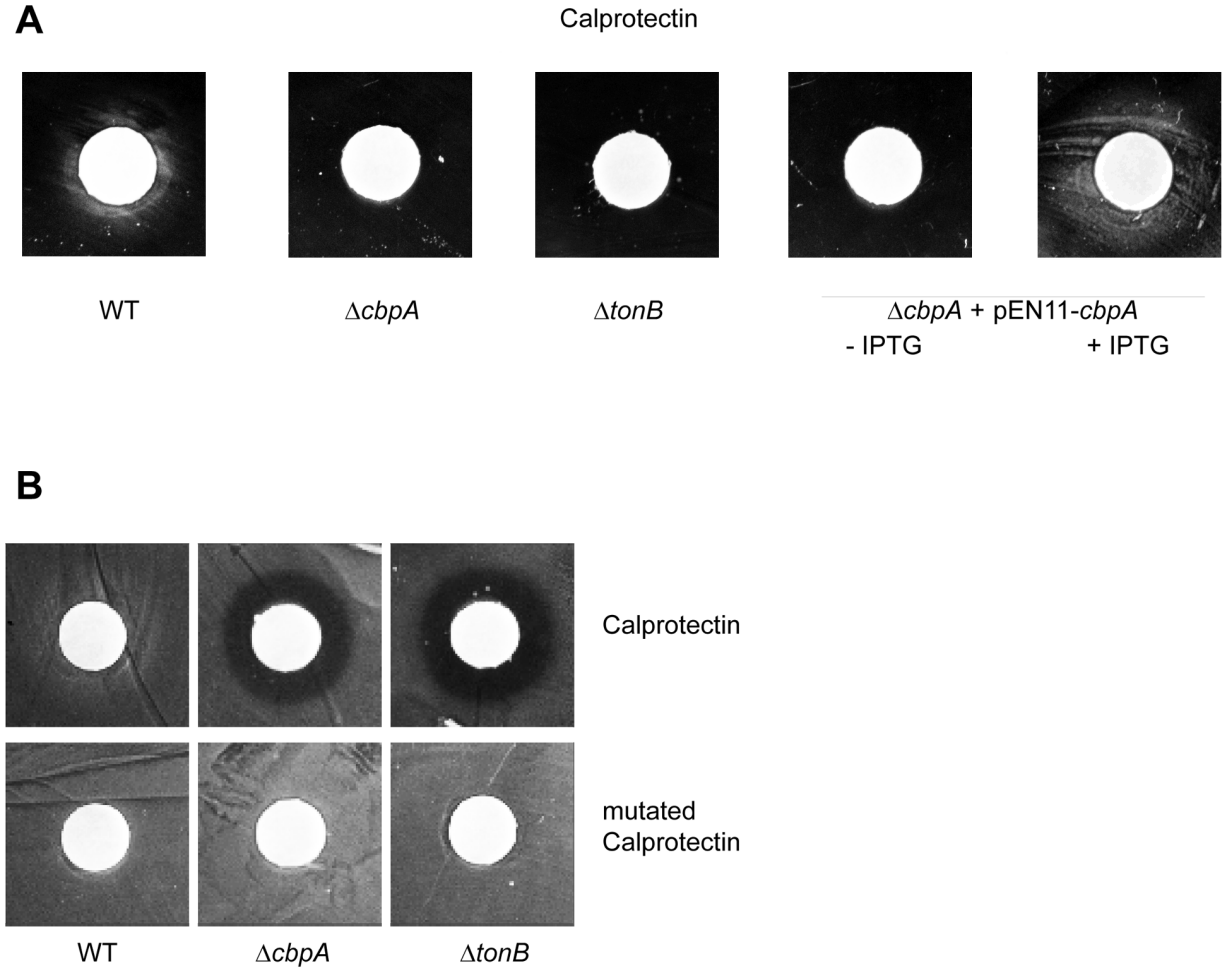
*N. meningitidis* can utilize calprotectin as a zinc source in a CbpA- and TonB-dependent manner. (A) Strain HB-1 (WT), its Δ*cbpA*- and Δ*tonB*-mutant derivatives, and the Δ*cbpA*-mutant derivative complemented with plasmid pEN11-*cbpA* were plated on RPMI agar plates supplemented with 10 µM FeCl_3_ as an iron source and with 1 µM TPEN to impose strict zinc limitation. In the case of the complemented strain, the medium was either supplemented or not with 100 µM IPTG as indicated. Filter discs containing 24.5 µg of calprotectin were placed on top of the plate and growth around the filter discs was evaluated after incubation overnight at 37°C. (B) Similar experiment as in panel A, except that the medium was not supplemented with TPEN. The filter discs contained either 24.5 µg calprotectin or 50 µg of a mutant form of calprotectin unable to chelate essential nutrient metals as indicated.

We next asked whether the capacity of CbpA-producing *N. meningitidis* to recruit calprotectin enables the cells to use it as a zinc source. To address this question, strain HB-1 and its *cbpA*- and *tonB*-mutant derivatives were inoculated on RPMI-medium plates supplemented with 1 µM TPEN to inhibit bacterial growth by zinc depletion. Then, filter discs containing calprotectin were placed on top of the plates. In this assay, calprotectin is expected to diffuse away from the filter disc and to stimulate the growth of bacteria that can use it as a zinc source. After overnight incubation of the plates, a growth zone was observed around the filter discs for the parental strain but not for the mutants (Figure 4A). The defect of the *cbpA* mutant to grow in the presence of calprotectin could be complemented by introduction of plasmid pEN11-*cbpA*, but only if expression of *cbpA* from the plasmid was induced with IPTG (Figure 4A). As a control, filter discs containing ZnSO_4_ were used, which stimulated the growth of all strains examined (Figure S2).

Whilst calprotectin can apparently stimulate the growth of CbpA-producing *N. meningitidis* under zinc deprivation, we anticipated that its zinc-sequestering activity would inhibit the growth of a *cbpA* mutant strain. To assess this possibility, the growth experiment described above on wild-type and mutant strains was repeated on plates not supplemented with TPEN. Growth of the wild-type strain was not inhibited but appeared even slightly enhanced around the calprotectin-containing filter discs (Figure 4B). In contrast, growth of both the *cbpA* mutant and the *tonB* mutant was severely affected as evidenced by a clear zone of growth inhibition around the discs (Figure 4B). This growth-inhibitory effect of calprotectin can be attributed to its nutrient metal-chelating properties, since a mutant form of calprotectin that cannot chelate Zn^2+^ and Mn^2+^ [16] did not inhibit the growth of the mutant strains (Figure 4B). Together, these experiments demonstrate that *N. meningitidis* can evade calprotectin-mediated nutritional immunity by using calprotectin as a zinc source via a mechanism that requires the outer-membrane receptor CbpA and the TonB complex.

### CbpA is universally expressed among meningococcal strains

**Figure 5:**
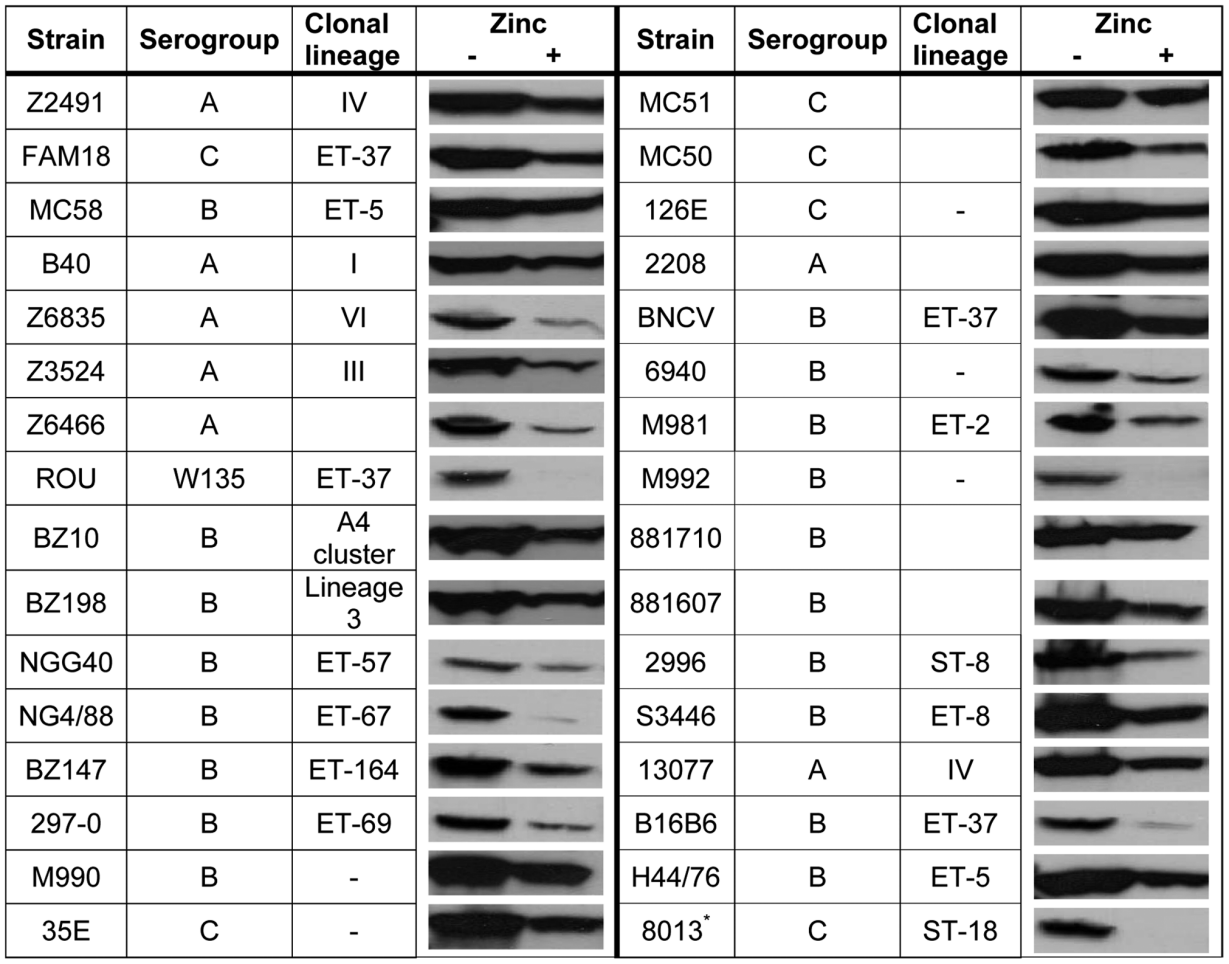
CbpA synthesis in a variety of meningococcal isolates. Various meningococcal isolates from our laboratory collection were grown in RPMI medium either supplemented or not with 0.5 µM ZnSO_4_ as indicated. After overnight growth, whole cell lysates were analyzed by Western blotting using an antiserum directed against CbpA. Where available [47], serogroups and clonal lineages of the strains are indicated. -, the strain was typed by Multi-Locus Enzyme Electrophoresis but could not be assigned to a specific clone.

The expression of many cell-surface-exposed proteins in *N. meningitidis* is prone to phase variation due to slipped-strand mispairing at short nucleotide repeats [17]. Inspection of the nucleotide sequence of *cbpA* and its promoter region did not reveal evidence for the presence of such repeats. Furthermore, the *cbpA* gene was found in all available genome sequences of *N. meningitidis* strains and the encoded protein showed high sequence conservation (Figure S3). Most of the variation is located in a small region between amino-acid residues 270–294 (Figure S3), which likely corresponds to a cell-surface-exposed loop of the protein that one anticipates, would be prone to immune selection. To further evaluate the conservation of the expression of *cbpA*, a series of strains was grown in RPMI medium either supplemented or not with 0.5 µM ZnSO_4_. Western blot analysis of whole cell lysates showed that the protein is expressed in all strains examined and that its expression is regulated by zinc availability (Figure 5). The ubiquitous presence of CbpA in all strains examined and the lack of phase variation suggest a vital role for the protein when the bacteria reside in the host.

## Discussion

Transition metals are essential nutrients for microbial growth. They play important structural and catalytic roles in many proteins. Nutritional immunity is a first line of defense, by which vertebrate hosts restrict the growth of microbial invaders by withholding them essential nutrients such as iron. Iron is sequestered in the human host by the iron-transport and -storage proteins transferrin and lactoferrin. The efficient acquisition of iron within the nutrient-restricted environment of the host is an essential virulence factor and has been studied extensively in many pathogens. Bacteria often respond to iron limitation by the production and secretion of iron-chelating compounds known as siderophores. Alternatively or in addition, they can directly access the host's iron resources such as heme, hemoglobin, haptoglobin, transferrin and lactoferrin. In all cases, iron acquisition from these resources requires a specific receptor in the outer membrane and the TonB complex that couples the energy of the electrochemical gradient across the inner membrane to the transport process in the outer membrane [1], [2], [6].

Only recently, it has become clear that nutritional immunity extends beyond iron deprivation to other transition metals including zinc and manganese [4], [18]. Amongst other mechanisms, these metals are sequestered in the human host by calprotectin, which is produced upon infection as a part of the innate immune response [19], [20]. Calprotectin is a heterodimer composed of S100A8 and S100A9, two members of the large S100 family of calcium-binding proteins implicated in defense against infection. It has two high-affinity binding sites, both of which bind Zn^2+^ whilst only one of them binds Mn^2+^ [4], [16]. By binding nutrient metal ions, calprotectin has demonstrated antimicrobial activity against many microorganisms including *E. coli*, *Acinetobacter baumannii*, *Borrelia burgdorferi*, *Staphylococcus aureus*, *Listeria monocytogenes*, and *Candida albicans* [4], [20]–[26]. Accordingly, the production of calprotectin for example in the inflamed intestine or the lungs of animal models has been shown to necessitate the expression of efficient zinc-acquisition systems for bacterial virulence and interbacterial competition [20], [27], [28]. Remarkably, *N. meningitidis* appears to use this defense mechanism of the host to its own benefit. We demonstrated here that the growth of this bacterium in a zinc-restricted environment is not inhibited but even stimulated by the presence of calprotectin. *N. meningitidis* produces an outer membrane receptor, CbpA, which enables it to use calprotectin as a zinc source. CbpA binds calprotectin more strongly when loaded with nutrient metals, which may facilitate the release of the ligand from the receptor after it has delivered its cargo to the bacterial cell. The acquisition of zinc from calprotectin by the meningococcus requires the TonB complex. In these respects, the acquisition of zinc parallels the use of transferrin or lactoferrin as iron sources [29].

In agreement with the role of CbpA in zinc acquisition is the observation that its production is induced under zinc limitation and under control of the transcriptional repressor Zur. These results are consistent with recent transcriptome analyses [30]. In contrast, *cbpA* expression appeared unaffected by the iron-responsive repressor Fur [31] consistent with the observed lack of regulation by iron availability (Figure S1). It will be interesting to investigate whether the meningococcus can use calprotectin also as a source of manganese. Consistent with such a role is the observation that loading of calprotectin with Mn^2+^ ions, like with Zn^2+^ ions, stimulated its binding to CbpA-expressing *N. meningitidis* cells (Figure 3D). However, unlike Zn^2+^ ions, Mn^2+^ ions in the low µM range did not repress CbpA synthesis (Figure 1B), suggesting a primary role for CbpA in the utilization of calprotectin as a zinc source. In contrast, a recent report suggested that growth inhibition of *S. aureus* by calprotectin was primarily related to its Mn^2+^-sequestering capacities [32]. In this respect, it is worth noting that an important aspect of the antibacterial activity of calprotectin against *S. aureus* in vivo resides in its capacity to inhibit the bacterial superoxide defenses, thereby enhancing the effectiveness of neutrophil oxidative burst [16]. *S. aureus* produces two superoxide dismutases, SodA and SodM, which are both Mn-dependent enzymes. In contrast, *N. meningitidis* produces a periplasmic superoxide dismutase, SodC, which is a Zn- and Cu-cofactored enzyme and has been shown to be implicated in protection against exogenous superoxide and in virulence in a mouse model of infection [33]. Thus, either the Mn^2+^- or the Zn^2+^-sequestering capacity of calprotectin might be more important, dependent on the target invading pathogen.

Previously, we have shown that *N. meningitidis* responds to zinc limitation by inducing the expression of another TonB-dependent receptor ZnuD, which may mediate the transport of free zinc [10]. Zinc-limitation-inducible expression of the genes for putative receptors has also been demonstrated recently in *A. baumannii* [20] and in the environmental bacteria *Pseudomonas protegens* [34] and the cyanobacterium *Anabaena* [35], demonstrating that zinc deprivation is an issue also for bacteria living in the environment. The ligands of these receptors have not yet been identified. BLAST searches at NCBI (results not shown) revealed the presence of CbpA homologs (>90% sequence identity) not only in meningococci but also in other *Neisseria* spp., including *N. gonorrhoeae* and the commensal *N. lactamica*. Often, these proteins are designated heme-utilization protein Hup. Hup is a protein with such function from *Haemophilus influenzae*, which shows sequence similarity (∼53% identity) to CbpA [36]. However, Turner et al. failed to demonstrate a role for CbpA (TdfH) in heme utilization [5]. These negative experimental data and the lack of responsiveness of *cbpA* expression to iron availability make an addition role of CbpA in heme utilization unlikely. This illustrates the danger of assigning functions to Tdf members merely based on sequence similarity. It seems likely that also other pathogens contain functional CbpA homologs that bind calprotectin. Their identification in genome sequences will be assisted by the identification of amino acids in CbpA that are involved in ligand binding and should therefore be conserved; this is our next aim. Also, it seems likely that some pathogens use other members of the S100 family as source of nutrient metals, such as psoriasin (S100A7), which binds Zn^2+^ [37], and calgranulin C (S100A12), which binds both Zn^2+^ and Cu^2+^ [38]. Plate assays such as those illustrated in Figure 4 may help to identify these pathogens. Clearly, with respect to new substrates of the Tdf members, we are currently probably only seeing the tip of the iceberg [39].

Altogether, studying the response of microorganisms to deprivation of transition metals other than iron is a rapidly expanding field, which will likely uncover many new interactions between pathogens and their hosts. In addition, these studies might reveal new strategies to combat these pathogens. We have recently demonstrated that ZnuD is an excellent candidate for the development of a broadly cross-protective vaccine against *N. meningitidis* [10], [40]. Here, we demonstrated that the CbpA protein was produced in all meningococcal strains examined indicating that its expression, unlike that of many other surface-exposed proteins of *N. meningitidis*, is not prone to phase variation. Therefore, and because it probably is an important virulence factor, CbpA may represent another interesting candidate for inclusion in such vaccine.

## Materials and Methods

### Bacterial strains and growth conditions

*N. meningitidis* strain HB-1 is an unencapsulated derivative of strain H44/76 [41]. Its *zur*- and *tonB*-mutant derivatives have been described [10]. Strain CE1523 is an unencapsulated *porA* mutant derivative of H44/76 [10]. Unless otherwise stated, *N. meningitidis* strains were grown at 37°C in candle jars on GC agar (Oxoid) plates containing Vitox (Oxoid) and antibiotics when appropriate (kanamycin, 150 µg/ml; chloramphenicol, 5 µg/ml). Liquid cultures were grown in TSB (Difco) or in RPMI (Sigma) at 37°C with shaking. The *E. coli* strains DH5α and TOP10F′ (Invitrogen), which were used for routine cloning, were grown on LB medium supplemented, when required, with 100 µg/ml ampicillin, 50 µg/ml kanamycin, or 25 µg/ml chloramphenicol.

### Constructions of mutants and plasmids

To knock out the *cbpA* gene on the chromosome, a DNA fragment upstream of this gene was amplified by PCR with primers P1TdfHEcoRI (5′-TGGGAATTCAGAACGTAAAATC-3′) and P2TdfHSalI (5′-CCTTGACGTCGACATCTTCC-3′) using chromosomal DNA from strain HB-1 as the template. Similarly, a DNA fragment downstream of the gene was amplified with primers P3TdfHSalI (5′-AAAGCGTGTCGACCAATTTTC-3′) and P4TdfHEcoRI (5′-GGGAATTCAGTTTTTTGAGT-3′). The fragments were cloned into pCRII-TOPO (Invitrogen) and joined together into one plasmid using the AccI sites that were introduced via the primers and the SpeI and XbaI sites in the vector. The resulting plasmid was designated pCRII-Δ*cbpA*. A kanamycin-resistance gene cassette was amplified from pKD4 [42] with primers P1 (5′-GTCGACGGATCCGTGTAGGCTGGAGCTGCTTC-3′) and P2 (5′-GTCGACGGATCCATGCCGTCTGAACATATGAATATCCTCCTTA-3′), the latter containing a neisserial DNA uptake sequence. Using the AccI sites that were introduced via the primers, the PCR product was inserted into the AccI site of pCRII-Δ*cbpA*. A PCR product containing the gene-replacement construct was amplified from pCRII-Δ*cbpA* with primers P1TdfHEcoRI and P4TdfHEcoRI and used to transform strain HB-1 as described [43] to generate the mutant strain designated HB-1Δ*cbpA*. Strain HB-1Δ*misRS* with a deletion of the *misRS* operon was constructed via a similar approach. In this case, the primer pairs used to amplify the upstream and downstream DNA fragment were P5MisREcoRI (5′-TCGTAGAATTCGCCCTGCCG-3′)/P6MisRSalI (5′-CAAGTCGACTACATCGTACTGCC-3′) and P7MisSSalI (5′-AACGCCGTCGACTACAGTCCC-3′)/P8MisSEcoRI (5′-GCGGATGGCGAATTCGGCGGTGT-3′), respectively.

To obtain the complementing plasmid pEN11-*cbpA*, a DNA segment encoding the mature part of CbpA was amplified by PCR from genomic DNA of strain H44/76 with phosphorylated primers IG-TdfH STOP rev (5′-CTTGGAGCATGCCTGCAGTTAAAACTTGTAGCTCATCGTCATC-3′) and IG-TdfH prot mature sens (5′-GAAGATGCAGGGCGCGCGGGC-3′). The PCR product was digested with PstI and cloned in a vector fragment obtained by PCR from pRIT16860 with primers IG-TdfI SS CPCR (CGCTTGGGCGAGGAGGGGTG) and TDFI_ND13 (CCGGCGACTATGTACGAGGCCG) that was also digested with PstI. pRIT16860 is similar to pEN11-*znuD* [10] but contains a kanamycin-resistance marker. The resulting plasmid, pRIT16864, contains a chimeric gene consisting of DNA fragments encoding the signal sequence of ZnuD and the mature part of CbpA and is cloned behind the *lac* promoter. To further improve *cbpA* expression, a DNA fragment containing the 3′ part of the *cbpA* gene including the transcriptional terminator was amplified from genomic DNA of strain HB-1 with primers TdfH-term-BspHI (5′-ATTCATGATTGGCATAGGCTTGCGGC-3′) and TdfH-Nde-U (5′-TTGAGGAACATATGAGATCT-3′) and cloned into pCRII-TOPO. A 2.1 kb SalI-NsiI fragment of the resulting plasmid was ligated into SalI-PstI restricted pRIT16864, yielding pRIT16864-term. Next, an NdeI-BspHI fragment of pRIT16864-term was ligated into NdeI-BspHI restricted pEN11-Imp [43] yielding pEN11-*cbpA*.

### Western blotting and antibodies

Whole cell lysates were prepared by resuspending cell pellets in sample buffer. Proteins were separated by SDS-PAGE and transferred to nitrocellulose membranes (Protran) using a wet transfer system (Biorad) in 25 mM Tris-HCl, 192 mM glycine, 20% methanol. Membranes were blocked for 1 h in phosphate-buffered saline containing 0.1% Tween-20 and 0.5% non-fat dried milk (Protifar, Nutricia). Blots were incubated with primary antibodies in blocking buffer. Antibody binding was detected by using peroxidase-conjugated goat anti-guinea pig or anti-mouse IgG secondary antibodies (Biosource) and enhanced chemiluminescence detection (Pierce). Bands were quantified by calculating the sum of pixels in a predefined area matching the size of the corresponding band in the reference sample. Background values were calculated from an empty area on the blot and automatically subtracted from every signal. The calculations where done with the pixel quantification plugin (version 1.2/R. Rosenman) in Adobe Photoshop.

Antiserum against fHbp and monoclonal antibodies against FbpA were generously provided by GlaxoSmithKline (Rixensart, Belgium) and Peter ven der Ley (RIVM, Bilthoven, The Netherlands), respectively. The antiserum against CbpA was obtained by immunizing six Hartley guinea pigs (female, 5–8 weeks old) (Charles River) via the intramuscular route on days 0, 14, and 28 with 10 µM purified recombinant His-tagged CbpA formulated in a water in oil emulsion. Antiserum was collected on day 42. The calprotectin-specific mAb 27E10 [44] was purchased from Hycult Biotech.

### qRT-PCR

qRT-PCR was performed as described previously [10]. The *rmpM* transcript was used to normalize all data. Primers used for the *cpbA* transcript were TdfHqF (5′-TCGACCCTCAGGATATATTCA-3′) and TdfHqR (5′-GCCCGAGCTTTTATCTTGCTG-3′).

### Subcellular localization

Cells of strain CE1523 containing pEN11-*cbpA* were grown for 2 h in TSB after which 1 mM IPTG was either added or not. Growth was continued for 4 h after which the cells were harvested by centrifugation (20,000 *g*, 10 min) and OMVs were isolated by extraction with DOC (Acros Organics) as described [45].

To determine the cell-surface exposure of CbpA, intact cells of strain HB-1, grown to mid-log phase in TSB, were collected by centrifugation and resuspended in 0.5 ml of 10 mM Tris-HCl, 5 mM MgCl_2_, pH 7.6. After addition of proteinase K, the cells were incubated for 20 min at room temperature. Then, 2 mM phenylmethylsulfonyl fluoride was added, and the cells were collected by centrifugation and analyzed by SDS-PAGE and Western blotting.

### Calprotectin binding assays

Cells of strain HB-1Δ*cbpA* containing pEN11-*cbpA* were grown in TSB either supplemented or not with 100 µM IPTG (Fermentas) to an optical density at 550 nm (OD_550_) of 1.0. The cells from 1 ml culture were harvested by centrifugation for 3 min in a microfuge at 8,000 *g*, washed in Hank's balanced salt solution (HBSS) (#14025, Life Technologies) and incubated for 1 h in 1 ml HBSS either supplemented or not with 4 µg calprotectin, which was prepared as described [46]. The cells were washed three times in HBSS, resuspended in sample buffer and cell-bound calprotectin was detected by Western blotting with calprotectin-specific mAb 27E10.

For indirect immunofluorescence microscopy, cells were grown, harvested and washed as above and then incubated for 15 h in 1 ml HBSS either supplemented or not with 10 µg calprotectin. Next, the cells were washed three times in HBSS and incubated for 1 h at room temperature on a rotating wheel with HBSS supplemented with bovine serum albumin to prevent non-specific binding of the antibodies. Then, 1 µg of mAb 27E10 was added to the solution followed by incubation for 2 h at room temperature on a rotating wheel. The cells were washed three times in HBSS and incubated for 1.5 h with Alexafluor-594-conjugated goat anti-mouse IgG antiserum (Molecular Probes) diluted 1∶500. Finally, the cells were washed three times and resuspended in 100 µl HBSS. Aliquots of 5 µl were spotted on a glass slide and subjected to bright field and immunofluorescence microscopy using an Olympos AX70 microscope.

### Calprotectin utilization assay

Bacteria were grown on RPMI-agar plates supplemented with 10 µM FeCl_3_ and solidified with 0.7% agar. After overnight growth, the bacteria were scraped from the plates and resuspended in RPMI medium to an OD_550_ of ∼1. Of these bacterial suspensions, 200-µl samples were plated on RPMI-agar plates supplemented with 10 µM FeCl_3_ and, where indicated, with 1 µM TPEN (Sigma) and/or 100 µM IPTG. Filter discs spotted with 5 µl of solutions containing 4.9 mg/ml wild-type calprotectin, 10 mg/ml of a mutant form of calprotectin that cannot bind Zn^2+^ or Mn^2+^ [16], or 10 µg/ml ZnSO_4_ were placed on top of the plates, which were subsequently incubated overnight at 37°C in candle jars.

## Supporting Information

Figure S1 Expression of *cbpA* does not respond to iron availability. Strain HB-1 was grown in RPMI medium supplemented with FeCl_3_ at the concentrations indicated. Whole cell lysates were then analyzed by SDS-PAGE followed by immunoblotting using antiserum directed against CbpA. The CbpA bands were quantified relative to the first lane on the blot and relative expression levels are indicated underneath the lanes.
(TIF)

Figure S2 Strain HB-1 (WT) and its Δ*cbpA*- and Δ*tonB*-mutant derivatives, were plated on RPMI agar plates supplemented with 10 µM FeCl_3_ as an iron source and with 1 µM TPEN to impose strict zinc limitation. Filter discs containing 5 µl of 10 µg/ml ZnSO_4_ were placed on top of the plates and growth around the filter discs was evaluated after incubation overnight at 37°C.
(TIF)

Figure S3 Alignment of the CbpA proteins from various meningococcal strains for which complete genome sequences are available.
(PDF)
