# Supplementary figures and images for: Zinc Piracy as a Mechanism of Neisseria meningitidis for Evasion of Nutritional Immunity
Source: PLoS Pathog. 2013 Oct 31;9(10):e1003733. doi: 10.1371/journal.ppat.1003733 (PMC3814407; doi:10.1371/journal.ppat.1003733)

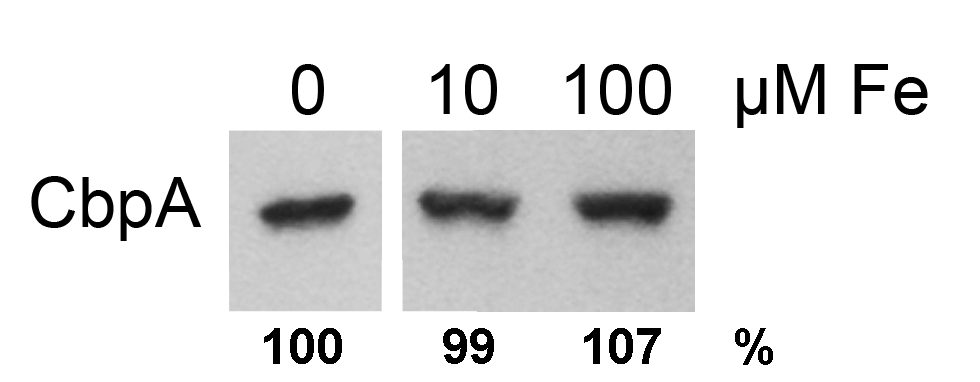

Supplement: Figure S1 — Expression of cbpA does not respond to iron availability. Strain HB-1 was grown in RPMI medium supplemented with FeCl3 at the concentrations indicated. Whole cell lysates were then analyzed by SDS-PAGE followed by immunoblotting using antiserum directed against CbpA. The CbpA bands were quantified relative to the first lane on the blot and relative expression levels are indicated underneath the lanes. (TIF) [file ppat.1003733.s001.tif]

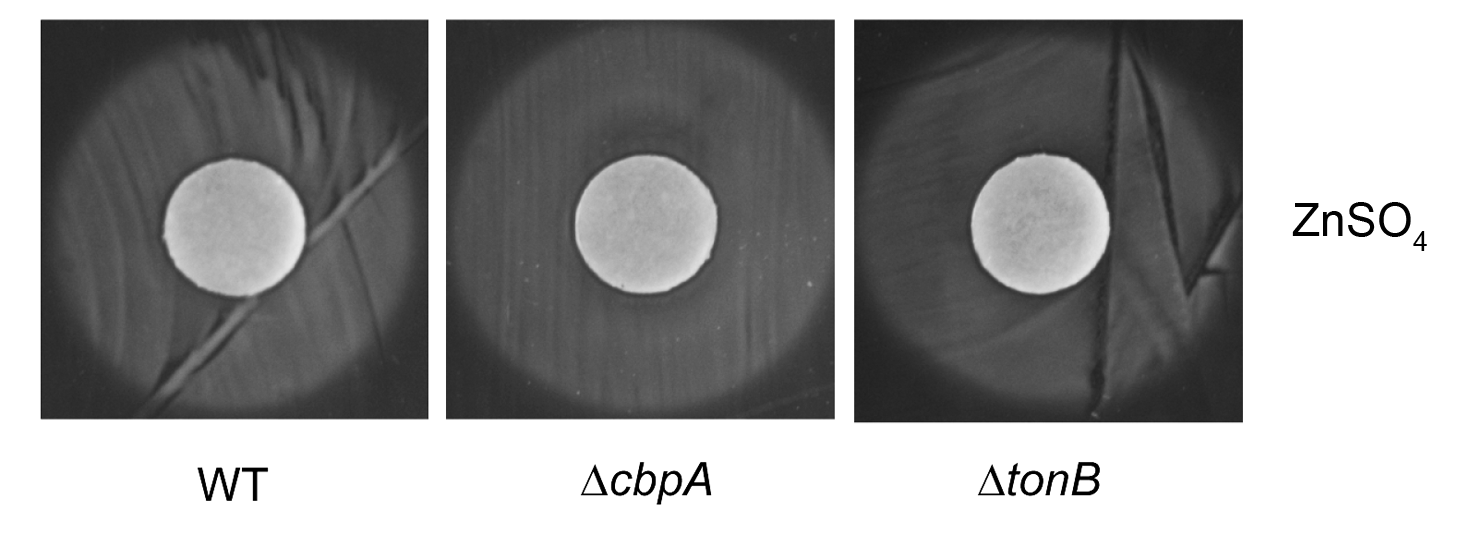

Supplement: Figure S2 — Strain HB-1 (WT) and its ΔcbpA- and ΔtonB-mutant derivatives, were plated on RPMI agar plates supplemented with 10 µM FeCl3 as an iron source and with 1 µM TPEN to impose strict zinc limitation. Filter discs containing 5 µl of 10 µg/ml ZnSO4 were placed on top of the plates and growth around the filter discs was evaluated after incubation overnight at 37°C. (TIF) [file ppat.1003733.s002.tif]
