# Supplementary material for: Zinc Piracy as a Mechanism of Neisseria meningitidis for Evasion of Nutritional Immunity
Source: PLoS Pathog. 2013 Oct 31;9(10):e1003733. doi: 10.1371/journal.ppat.1003733 (PMC3814407; doi:10.1371/journal.ppat.1003733)
Supplement: Figure S3 — Alignment of the CbpA proteins from various meningococcal strains for which complete genome sequences are available. (PDF) [file ppat.1003733.s003.pdf]

```
MC58          MRSSFRLKPICFYLMGVTLYHYSYAEDAGRAGSEAQIQVLEDVHVKAKRVPKDKKVFTDARAVSTR
H44/76        ..................................................................
O53442        .................M...H............................................
Z2491         .................M...H............................................
FAM18         .................M...H............................................
8013          .................M...H........T...................................
ALPHA14       .................M...H............................................
ALPHA153      .................M...H............................................
ALPHA170      .................M...H............................................
ATCC-13091    .................M...H............................................
MO1-240355    .................M...H............................................
G2136         .................M...H............................................
N1568         .................M...H............................................
MO1-240013    .................M...H........T...................................

MC58          QDIFKSSENLDNIVRSIPGAFTQQDKSSGIVSLNIRGDSGFGRVNTMVDGITQTFYSTSTDAGRAG
H44/76        ..................................................................
O53442        ..................................................................
Z2491         ..................................................................
FAM18         ..................................................................
8013          ..................................................................
ALPHA14       ..................................................................
ALPHA153      ..................................................................
ALPHA170      ..................................................................
ATCC-13091    ..................................................................
MO1-240355    ..................................................................
G2136         ..................................................................
N1568         ..................................................................
MO1-240013    ..................................................................

MC58          GSSQFGASVDSNFIAGLDVVKGSFSGSAGINSLAGSANLRTLGVDDVVQGNNTYGLLLKGLTGTNS
H44/76        ..................................................................
O53442        ..................................................................
Z2491         ..................................................................
FAM18         ..................................................................
8013          ..................................................................
ALPHA14       ..................................................................
ALPHA153      ..................................................................
ALPHA170      ..................................................................
ATCC-13091    ..................................................................
MO1-240355    ..................................................................
G2136         ..................................................................
N1568         ..................................................................
MO1-240013    ..................................................................

MC58          TKGNAMAAIGARKWLESGASVGVLYGHSRRSVAQNYRVGGGGQHIGNFGAEYLERRKQRYFVQEGA
H44/76        ..................................................................
O53442        ..................................................................
Z2491         ............................................................E....G
FAM18         ..........................................................Q....T.G
8013          ..........................................................Q....T.G
ALPHA14       ..........................................................Q....T.G
ALPHA153      ...............................................................D.Q
ALPHA170      ...............................................................D.Q
ATCC-13091    ..................................................................
MO1-240355    .................................................................G
G2136         ..................................................................
N1568         ..................................................................
MO1-240013    ...............................................................T.G
```

```
MC58         LKFNSDSGKWERDLQRQQWKYKPYKNYNN-QELQKYIEEHDKSWRENLAPQYDITPIDPSSLKQQS
H44/76       ..................................................................
O53442       ...........P.............K........................................
Z2491        ....N.......F.KSY.KT.W..QK.DAP........G...........................
FAM18        .....N...........F..TSY..Q.KDP.K.K....DQ..D.......................
8013         .....N...........F..TSY..Q.KDP.K.K....DQ..D.......................
ALPHA14      .....N...........F..TSY..Q.KDP.K.K....DQ..D.......................
ALPHA153     .....................PW..K.E.P....................................
ALPHA170     .....................PW..K.E.P....................................
ATCC-13091   .....................SW..K.E.P....................................
MO1-240355   .....N.......F..PY....W.QK..DP........G...........................
G2136        ..................................................................
N1568        ...........P...K.....N...K........................................
MO1-240013   .............F...F..H.L..K.DDP........GQ..D.......................

MC58         AGNLFKLEYDGVFNKYTAQFRDLNTKIGSRKIINRNYQFNYGLSLNPYTNLNLTAAYNSGRQKYPK
H44/76       ..................................................................
O53442       ..............................................S.A.................
Z2491        ..................................................................
FAM18        ..............................................S...................
8013         ..............................................S.A.................
ALPHA14      ..................................................................
ALPHA153     ..............................................S.A.................
ALPHA170     ..............................................S.A.................
ATCC-13091   ..............................................S.A.................
MO1-240355   ..................................................................
G2136        ..............................................S.A.................
N1568        ..............................................S.A.................
MO1-240013   ..................................................................

MC58         GSKFTGWGLLKDFETYNNAKILDLNNTATFRLPRETELQTTLGFNYFHNEYGKNRFPEELGLFFDG
H44/76       ..................................................................
O53442       ..................................................................
Z2491        ...........................S......................................
FAM18        ..................................................................
8013         ..................................................................
ALPHA14      ..................................................................
ALPHA153     ..................................................................
ALPHA170     ..................................................................
ATCC-13091   ..................................................................
MO1-240355   ..................................................................
G2136        ..................................................................
N1568        ..................................................................
MO1-240013   ..................................................................

MC58         PDQDNGLYSYLGRFKGDKLLPQKSTIVQPAGSQYFNTFYFDAALKKDIYRLNYSTNTVGYRFGGGE
H44/76       ..................................................................
O53442       ..................................................................
Z2491        ..................................................................
FAM18        ..................................................................
8013         ..................................................................
ALPHA14      ..................................................................
ALPHA153     ..................................................................
ALPHA170     ..................................................................
ATCC-13091   ..................................................................
MO1-240355   ..................................................................
G2136        ..................................................................
N1568        ..................................................................
MO1-240013   ..................................................................
```

```
MC58          YTGYYGSDDEFKRAFGENSPTYKKHCNRSCGIYEPVLKKYGKKRANNHSVSISADFGDYFMPFASY
H44/76        ..................................................................
O53442        .....S.EG.........................................................
Z2491         ...........................Q......................................
FAM18         .....S.EG..................P...L..................................
8013          ...........................Q......................................
ALPHA14       .......EG...........I.RE..DP...L..................................
ALPHA153      .....S.EG..................P...L..................................
ALPHA170      ......................RE...Q......................................
ATCC-13091    .....S.EG.............RE...P...L..................................
MO1-240355    .....S.EG.............RE...P...L..................................
G2136         ...........................P...L................................G.
N1568         ...........................P...L..................................
MO1-240013    ..................................................................

MC58          SRTHRMPNIQEMYFSQIGDSGVHTALKPERANTWQFGFNTYKKGLLKQDDTLGLKLVGYRSRIDNY
H44/76        ..................................................................
O53442        ..................................................................
Z2491         ..................................................I...............
FAM18         ..................................................................
8013          ..................................................I...............
ALPHA14       ..................................................................
ALPHA153      ..................................................................
ALPHA170      ..................................................................
ATCC-13091    ..................................................................
MO1-240355    ..................................................................
G2136         ..................................................................
N1568         ..................................................................
MO1-240013    ..................................................................

MC58          IHNVYGKWWDLNGDIPSWVSSTGLAYTIQHRNFKDKVHKHGFELELNYDYGRFFTNLSYAYQKSTQ
H44/76        ..................................................................
O53442        .............N.....N..............................................
Z2491         .............N....................................................
FAM18         ..................................................................
8013          .............N....................................................
ALPHA14       .............N....................................................
ALPHA153      ..................................................................
ALPHA170      .............N....................................................
ATCC-13091    ..................................................................
MO1-240355    ..................................................................
G2136         .............N....................................................
N1568         .............N....................................................
MO1-240013    ..................................................................

MC58          PTNFSDASESPNNASKEDQLKQGYGLSRVSALPRDYGRLEVGTRWLGNKLTLGGAMRYFGKSIRAT
H44/76        ..................................................................
O53442        ..................................................................
Z2491         ..................................................................
FAM18         ..................................................................
8013          ..................................................................
ALPHA14       ..................................................................
ALPHA153      ..................................................................
ALPHA170      ..................................................................
ATCC-13091    ..................................................................
MO1-240355    ..................................................................
G2136         ..................................................................
N1568         ..................................................................
MO1-240013    ..................................................................
```

```
MC58          AEERYIDGTNGGNTSNFRQLGKRSIKQTETLARQPLIFDFYAAYEPKKNLIFRAEVKNLFDRRYID
H44/76        ..................................................................
O53442        ................V.................................................
Z2491         ..................................................................
FAM18         ..................................................................
8013          ..................................................................
ALPHA14       ................V.................................................
ALPHA153      ................V.................................................
ALPHA170      ................V.................................................
ATCC-13091    ................V.................................................
MO1-240355    ................V.................................................
G2136         ..................................................................
N1568         ..............RDV.................................................
MO1-240013    ..................................................................

MC58          PLDAGNDAATQRYYSSFDPKDKDEDVTCNADKTLCNGKYGGTSKSVLTNFARGRTFLMTMSYKF
H44/76        ................................................................
O53442        ........................E.......................................
Z2491         ........................E....D.N.........................I......
FAM18         ...............................-G...............................
8013          ................................................................
ALPHA14       ........................E....D.N.........................I......
ALPHA153      ................................................................
ALPHA170      ...............................-G........................I......
ATCC-13091    ........................E....D.N.........................I......
MO1-240355    ........................E.......................................
G2136         ...............................-G...............................
N1568         ........................E....D.-G........................I......
MO1-240013    ...............................-G........................I......
```
